# The role conflict-burnout-depression link among Chinese female health care and social service providers: the moderating effect of marriage and motherhood

**DOI:** 10.1186/s12889-022-12641-y

**Published:** 2022-02-04

**Authors:** Guanghuai Zheng, Xinshu Lyu, Li Pan, Anna Chen

**Affiliations:** 1 Department of Social Work, Central China Normal University, NO.152 Luoyu Road, Wuhan, Hubei 430079 P. R. China; 2 School of Social Development and Public Policy, Beijing Normal University, Xinjiekouwai St, Haidian District, Beijing, 100875 P. R. China

**Keywords:** Depression, Organizational role conflict, Work-family conflict, Women, Health care and social service providers, Exhaustion, Cynicism

## Abstract

**Background:**

Women with depression disorder outnumber men, and health care and social service providers are mostly female. Drawing on conservation of resources (COR) theory, this study aims to examine the association between role conflicts and depression among health care and social service providers, and further investigate the mediating effect of burnout, as well as the moderating effect of marital status and motherhood.

**Methods:**

The data come from the baseline of the ‘China Social Work Longitudinal Study’ conducted in 2019, which contains 1,219 female social workers who reported work-family conflict. The five items of the scale in our model were extracted from the existing literature to ensure the construct validity of potential variables, and confirmatory factor analyses (CFAs) were also conducted to ensure the validity and reliability of the scale. Descriptive analyses and correlation analyses were performed with SPSS 24, while the path analysis was conducted using Amos 24. The moderating effects of marital status and motherhood were further tested using multiple-group analyses.

**Results:**

Female health care and social service providers experienced a high level of depression. Work-to-family conflict (WFC), family-to-work conflict (FWC), and organizational role conflict (ORC) were significantly and positively associated with female social workers’ depression. Exhaustion and cynicism fully mediated the effects of ORC on depression and partially mediated the effects of WFC on depression. In addition, FWC had only a direct effect on depression. A multiple-group analysis further indicated that both marital status and motherhood status may have played a moderating role in the conflict-burnout-depression link and that being unmarried and having no child were risk factors for depression in female health care and social service providers.

**Conclusions:**

Marriage and motherhood have both negative and positive effects on the depression of female health care and social service providers. This suggests that marriage and motherhood may act as a form of “family clientelism” for female health care and social service providers who marry and have children.

## Introduction

In the rapidly changing work environment influenced by economic globalization and the rise of neoliberal policies and managerialism, health care and social service providers (e.g., nurses, teachers, physicians, psychologists, and social workers) can experience higher rates of depression than the general population [1, 2]. According to studies conducted in mainland China, the prevalence of depressive symptoms among health care and social service providers was estimated to be 25.9-38% [3, 4]. Depression has been found to be associated with the professional attitudes (e.g., turnover intentions, job satisfaction, organizational commitment) of health care and social service providers and is associated with impaired quality and security of the service provided [5]. As the majority of health care and social service providers are women and evidence show that female health care and social service providers are at risk of a higher level of depression than male health care and social service providers [6], investigating females’ experiences may provide insights into the nature of depression for people working in the social service sector.

Empirical evidence has consistently indicated that women with depression disorder outnumber men at a ratio of 2:1 [7]. Following Phyllis Chesler’s influential study *Women and Madness* in suggesting that gender is embedded in the construction of the concepts of madness and mental disorder, feminist scholarship has argued most successfully in its accounts of why women may be depressed. Feminist paradigms document women’s depression as a function of gendered expectations that are inherent in their stressful social roles, including marriage, motherhood, employment and so forth [8, 9]. Previous research demonstrates that more than one-third of the gender differences in depression among medical workers are explained by work-family conflict [10]. In addition to workload, employed women have to address family needs (e.g., child care, housework), resulting in them genuinely suffering more distress than men [11]. We adopt a feminist perspective to explore the relationship between role conflicts and female health care and social service providers’ depression.

Furthermore, previous studies have highlighted that burnout is a mediating state that accelerates negative mental health effects and leads to depression [12]. Burnout is a long-term psychological response to chronic emotions and stress related to work, manifested by emotional exhaustion, cynicism (personality disintegration), and reduced personal accomplishment [12]. Empirical research has suggested that health care and social service providers may experience higher levels of role conflicts and resultant burnout than general occupational groups [13]. However, recent studies have noted that the relationship between work-family conflict and burnout is rarely researched in social services [14]. This paucity leaves a research gap that we attempt to address by examining whether burnout mediates role conflicts in depression.

In addition, there is a small but powerful literature on the relationship between family stress and depression in working people [15]. Marriage and motherhood have been put forward as risk factors for women’s depression [9, 16], with young married women with small children deemed to be at particularly high risk [17]. In addition, researchers are now examining whether family resources are significantly associated with reduced depression [18]. A number of studies report that marriage acts as a protective factor to buffer psychological distress [19], with divorced or separated individuals at higher risk of depression than married individuals [20]. Therefore, marriage and motherhood as moderators should be considered to establish mechanisms that link the role conflicts of female health care and social service providers to the construction of their depression.

Conservation of resources (COR) theory has been widely adopted in burnout and distress research. COR theory states that individuals are motivated to protect the resources they value, but when such efforts fail, depression may occur as employees feel incapable of coping with high demands [21, 22]. Drawing on COR theory, this article aims to take broad theoretical and empirical strokes examining the effect of role conflicts on female health care and social service providers’ depression, whether burnout as a psychological response to stress related to work mediates the relationship between role conflicts and depression, and how family resources (marriage and motherhood) moderate the conflict-burnout-depression link among female health care and social service providers. We used data from the China Social Work Longitudinal Study (CSWLS) in 2019 [23], a large-scale, continuous sampling survey and research project in China targeting the development and trends of social work, to provide insights into the social service industry. Previous research shows that social workers report twice the level of distress as those in comparable occupational roles (e.g., psychiatrists) [13, 24]. In mainland China, social workers play major roles in community integrated social services; e.g., the Guangdong Civil Affair Department established 407 social work stations in 2017-2020, and each station enrolled 6 social workers [25]. To date, most research on the mental health of health care and social service providers has focused on role conflicts within organizations, ignoring the perspective of work-family interference. By incorporating multiple role conflicts and a feminist perspective into our model, we provide a more nuanced understanding of depression among female health care and social service providers, who make up the majority of health care and social service providers.

## Literature review

### From conflict to understanding depression: theoretical perspective

#### Role conflict and women’s depression

Conceptually, a role originates from expectations about behaviour for a position in a social structure, and role conflicts occur as a result of incompatible behavioural expectancies of role-relevant others [26]. Given the widespread prevalence of depression and its accompanying negative consequences, the relationship between role conflicts and depression has witnessed growing academic interest [27]. Drawing on COR theory, individuals are motivated to protect the resources they value, but when such efforts fail, employees may experience depression, as they feel incapable of coping with high demands [21, 22]. Previous research has pointed out the tension between the resources underlying role conflicts, as participation in a specific role will lead to more interference among multiple roles due to resource competition [22, 28].

The current study also suggests the need for a nuanced role conflict-depression model, which covers various kinds of role conflicts. Generally, there are two sorts of role conflict: inter-role conflict and intra-role conflict. Inter-role conflict is a clash between the multiple requirements of two or more roles, and intra-role conflict involves incompatible demands within the same role [26]. Inter-role conflict includes family-to-work conflict (FWC), which involves stress caused by the family responsibilities interfering with work-related responsibilities, and work-to-family conflict (WFC), which occurs when work-related stress interferes with family-related responsibilities [28]. Organizational role conflict (ORC) is a typical type of and well documented intra-role conflict, that involves attempting to meet the distinct expectations of multiple authorities in an organization [29, 30].

It is noteworthy that the role conflict perspective is important for understanding female depression [30]. Working women suffer from greater work-family conflict than men, as traditional gender roles assume that family roles are primary for women and that work roles are secondary [19]. Women often face higher ORC because gender stereotyping has excluded women from professional work and arranges the bulk of the low-level tasks for them [31]. Therefore, to obtain a precise understanding of depression in working women, FWC, WFC and ORC should be included in studies of women’s depression.

#### Role conflict and depression of female health care and social service providers

The importance of studying the antecedents of depression among health care and social service providers is demonstrated in the prevalence of depression among them and its negative consequences. Previous studies have provided evidence that health care and social service providers suffer from role conflicts, which are related to their mental health problems. First, health care and social service providers are often expected to fulfil a caregiver role in their family as well, which means more family life responsibilities, leading to work-family conflict and depression [18]. Second, it has been well documented that health care and social service providers face ORC pressure, especially in the era of neoliberal reform [29]. Conflict requests from clients, governments, and organizations contribute to depressive symptoms [1].

Scholars are increasingly interested in role conflict in cultural contexts different from western individualism [32, 33]. According to the sociologist Xiaotong Fei, Chinese society has emerged as a fundamentally rational society with a hierarchy of social ranks [34]. Clan culture in China emphasizes male dominance in the family [35]. Influenced by this cultural dimension of male dominance, Chinese women voluntarily and involuntarily combine paid work with domestic work [36, 37], leading to a higher level of work-family conflict than men experience. Since the enforcement of the universal two-child policy in 2016, more female workers have found themselves caught in the dilemma of whether to raise a child or be promoted, which exacerbates their WFC and worsens their mental health [38]. The masculine culture also shows in the workplace, where Chinese female social workers undertake more emotional labor than male social workers, causing organizational role conflict and psychological problems among them [39]. ORC has also been shown to predict the emergence of depressive symptoms in Chinese female nurses [40]. Therefore, we propose the following:

**H1.** FWC is directly and positively associated with the depression of female health care and social service providers.

**H2.** WFC is directly and positively associated with the depression of female health care and social service providers.

**H3.** ORC is directly and positively associated with the depression of female health care and social service providers.

### Mediation model development

#### The role conflict-burnout-depression link

While assessing the independent effects of role conflict is of primary importance, another promising direction for investigation is suggested by previous studies [41], i.e., whether burnout mediates the relationship between role conflicts and depression. According to COR theory, role conflicts can lead to burnout and trigger chronic mental disorders (e.g., depression) because the basic motivation to protect resources is threatened or denied [22]. Therefore, COR theory serves as a heuristic model for the role conflict-burnout-depression link.

Among the three dimensions of burnout, in line with COR theory and previous research, reduced personal accomplishment is a result of burnout rather than a distinct symptom, and role conflicts has a stronger relation with emotional exhaustion and cynicism than reduced personal accomplishment [42, 43]. A recent study also pointed out that exhaustion and cynicism are core components that affect social welfare workers in China, while personal accomplishment is not [44]. Therefore, we chose to limit our analysis to emotional exhaustion and cynicism in our model.

Previous investigations have provided evidence of the role of the conflict-burnout-depression link. Existing studies highlight that burnout is a mediating state that accelerates the negative effects of mental health (such as anxiety and drops in self-esteem) and leads to depression [12]. A meta-analysis focused on work-family conflict, and its various outcomes has shown a positive relationship between FWC, WFC and burnout [45]; a recent literature review pointed out that work-family conflict made employees feel less control and predicted higher burnout [46]. Regarding ORC, research evidence consistently supports its positive correlation with burnout, as both increased work demands and decreased work resources predict burnout [47].

#### The role of the conflict-burnout-depression link among health care and social service providers

Empirical evidence has revealed that emotional exhaustion and cynicism are prevalent among health care and social service providers [48], which also bring about a series of negative consequences, including depression and anxiety, poor-quality care, absenteeism and turnover [41]. Substantial research has focused on the relationships between FWC, WFC, ORC, burnout, and depression among service providers, with mixed results. First, previous findings suggest that ORC is correlated with health care and social service providers’ emotional exhaustion and cynicism [48]. An ORC-burnout-depression connection has also been found among physicians [41]. Second, previous research on service providers has pointed out that both WFC and FWC are related to burnout and found a stronger impact of the two on burnout than on depression [49]. Meanwhile, previous studies have pointed out gender differences in the role conflict-burnout relationship, as females report higher levels of burnout than men due to lower levels of decision latitude and self-esteem, as well as higher levels of work-family conflict [50]. A study on Chinese female service providers conducted by Wang et al. [51] pointed out that female service workers are more susceptible to WFC than male service workers, and both WFC and FWC were positively related to their emotional exhaustion and cynicism. Therefore, we propose the following:

**H4.** Emotional exhaustion mediates the relationship between role conflicts (H4a: FWC; H4b: WFC; H4c: ORC) and depression among female health care and social service providers.

**H5.** Cynicism mediates the relationship between role conflicts (H5a: FWC; H5b: WFC; H5c: ORC) and depression among female health care and social service providers.

### The moderating role of marriage and motherhood on female health care and social service providers’ depression

As mentioned above, a multiplicity of roles straddled does not always result in role conflicts but depends on whether a particular role provides resources and role requirement conflicts [19]. That is, a model that considers particular roles as well as both the benefits and conflicting needs of these roles may be necessary to predict the role conflict-burnout-depression link. In industrialized economies, the social roles of females are often organized as homemakers and the primary caretakers of children, while males are more likely to become primary family providers and to assume full-time roles in the paid economy [31]. To obtain a better understanding of the depression of female health care and social service providers, we tested two possible moderators of the associations in the model: (a) marriage and (b) motherhood.

#### Marriage

The heated debate about whether marriage is a “health hazard” for women turns on conflicting evidence of the relationship between marriage and depression. A recent review documented that married people tend to report a higher level of mental health, as they have more psychosocial resources, and unmarried people are more vulnerable to strain due to a lack of spouse support [52]. A previous study also indicated the emotional benefits of marriage for both men and women, suggesting that marriage is associated with enhanced mental health [53, 54]. For health care and social service providers, previous research has also reported that divorced, separated, and never-married social workers are more likely to report depression [1].

However, previous studies provide inconsistent evidence, indicating that marriages enhance depressive symptoms. Previous research on marital status has shown that marriage is a risk factor for female depression, as marital conflict directly leads to increased depression [55]. Similarly, a trickle of studies have revealed that married females experience a higher rate of role conflicts, resulting in job burnout and depression [28]. Since contradictory normative beliefs limit women’s roles to household tasks in marriage, married professional women have been found to suffer from greater incompatibility between marriage and career [28], which also results in increased burnout. Therefore, we propose the following:

**H6.** Marriage moderates the role conflict-burnout-depression model, as married female health care and social service providers are more likely to experience role conflict, burnout, and depression.

#### Motherhood

Contrary to the inconsistent findings with respect to the role of marriage and its relationship with depression, there is substantial evidence that the role of motherhood enhances working mothers’ depression. Previous studies have pointed out that depression is positively related to role conflict for women with child-care responsibilities [56]. Child-care responsibilities such as breastfeeding, physical care, and education associated with the mother’s role can interfere with working women’s work responsibilities and exacerbate their work-family conflict [28]. Meanwhile, some studies have documented that parenting is one of the most demanding and stressful life changes individuals face, often giving rise to depressive symptoms, and postpartum depression is a typical case [57]. A recent study on parental and job burnout in a Chinese context showed that both parenthood and job burnout have a strong positive correlation with depression [58]. Therefore, we propose the following:

**H7.** Motherhood moderates the role conflict-burnout-depression relationship, as female health care and social service providers with children are more likely to experience role conflict, burnout, and depression.

## Methods

### Participants and procedures

This study chose social workers as a representative of health care and social service providers because of their important role in current social service delivery in China and their characteristics of femininity, overwork, and low wages [39, 40]. To investigate our hypotheses, this study adopted data from the CSWLS in 2019 [23], which is the first large-scale, continuous sampling survey and research project in China targeting the development and trends of social work. The procedure for selecting the sample units consisted of four steps: (a) 56 municipalities of five strata as the first sampling unit; (b) social work agencies as the second sampling unit, which must be registered as private non-profit organizations before June 1, 2019; (d) individual social workers who had been working in social work agencies for more than three months as the third sampling unit; and (d) sample supplementation and replacement [23, 35]. Eventually, a total of 993 questionnaires from social work institutions and 6,785 questionnaires from social workers were obtained. Due to the data collection under the 4-round quality control, the overall data quality of this 2019 wave was good. According to the CSWLS database, the 4-round quality control included on-site inspection of questionnaire collection, review of local project implementation teams after questionnaire collection, acceptance of questionnaires by supervisors dispatched from each region, and re-inspection of project teams before questionnaire entry [23, 35].

Specifically, among the questionnaires, 979 institutional and 6,776 individual questionnaires were valid (effective response rate = 98.59% and 99.87%, respectively [23];). It finally released survey data for 979 social work agencies and 5,965 social workers (excluding 811 civil affairs and medical social workers). For the social workers, the questionnaires had multiple modes and spanned multiple domains covering the social workers’ personal, family, and professional situations and statuses, including work-related stress and problems. Among the participants, the average age was 30.44 years; 4,714 (79%) were female; 3,611 (60.5%) held at least an assistant social work license; 3,330 (56%) had a bachelor’s degree; and 1,432 (24%) were members of the Communist Party of China.

Consistent with our research perspective, we focus on role conflicts to understand female health care and social service providers’ depression. We first focused on the 4,714 female social workers. Among them, we found that a total of 1,219 social workers completed scales of work-family conflict. Therefore, for the purpose of this study, we used data collected from 1,219 female social workers who reported role conflicts. Of the total respondents, the mean age was 31.5 years (SD = 8.4), 47% were married (*n* = 573), 37.7% had at least one child, 58.8% had a bachelor’s degree or beyond (*n* = 717), and 18% had a social work license (*n* = 220). They were all full-time social workers, and their length of experience in social work (job tenure) is 6.45 years.

### Measurement

#### Depression

In the CSWLS, depression was measured by 20 items on the Center for Epidemiological Studies-Depression scale (CES-D) [59], which queries respondents’ daily lives in the past week and invites them to report their situation over the past week to answer each question. Respondents are asked how often they could not get going, felt sad, enjoyed life, felt lonely, were happy, slept restlessly, felt that everything took effort, and felt depressed; these items were rated 0 (none of the time), 1 (1-2 days), 2 (3-4 days), or 3 (5-7 days). We consider depression to be a continuous phenomenon, with higher scores indicating a more depressed mood. In addition, we further added the scores of the 20 items to get the total rough score, the rough score was then multiplied by 1.25, and the integer was used as the standard score of their depression. The mean depression score of our female social workers was 37.96 (SD = 11.95; according to the normality of the Chinese sample, the cut-off point of depression was 53 points for mild depression). The scale’s overall Cronbach’s alpha coefficient was 0.932.

#### Independent variables and mediators

Role conflict refers to incompatible demands placed upon a person in relation to their different social roles [60, 61]. Survey items were drawn from the literature to ensure the construct validity of the three core independent variables—WFC, FWC, and ORC—and the two mediators—exhaustion and cynicism. First, we measured female social workers’ WFC using 3 items and measured FWC using 4 items [62, 63], which were modified to fit the context in China. Here, the two kinds of conflicts involve the degree of conflict the female social workers attributed to their struggles in the family and work arenas, which they rated on a five-point Likert scale (1-*strongly disagree* to 5-*strongly agree*). Sample items for WFC include “When I get home, the worries or problems at work still haunt me” and “Busy work makes me have no time to participate in family activities”, while those for FWC include “Personal or family problems distract me from my work” and “Family pressure makes me irritable at work”. Then, we adapted the role conflict scale [60], a self-report questionnaire consisting of nine items, from the Chinese version [61] to measure ORC the degree to which female social workers rated their job duties and responsibilities on a five-point Likert scale (1-*strongly disagree* to 5-*strongly agree*). Sample items for ORC include “I lack sufficient resources to complete the tasks assigned by my superiors” and “I am working on two project teams with different working styles at the same time”. High scores on the three measurements indicates a high level of role conflicts. The scale’s overall Cronbach’s alpha coefficients for WFC, FWC, and ORC were 0.817, 0.861 and 0.814, respectively. Finally, we used the Chinese version of the burnout scale, which was adopted from the MBI-GS [64] and validated in Chinese social workers, to measure cynicism and exhaustion [44]. According to previous research, reduced personal accomplishment is a result of burnout rather than a distinct symptom [43]. Therefore, we chose to limit our analysis to emotional exhaustion and cynicism in our model, similar to what has been done by other authors. All items are related to burnout in a work setting. The questionnaire is scored on a 7-point Likert scale from 0 (never) to 6 (daily). High scores on the two measurements indicates a high level of burnout. The scale’s overall Cronbach’s alpha coefficients for exhaustion and cynicism were 0.910 and 0.745, respectively.

#### Common method bias and confirmatory factor analysis

For statistical control, we used Harman’s one-factor test. When loading the models’ six variables into an exploratory factor analysis, the first emerging unrotated factor with an eigenvalue over one, accounted for 45.154% of the overall variance. This was lower than the 50% threshold suggested by Podsakoff [65]. In addition, we conducted a marker variable technique suggested by [66] to determine whether common method variance threatened the results. We used a variable of ‘the exact length participated in the survey’ (continuous variable) as the marker variable. That variable was theoretically unrelated to all the latent variables in our study. Correlation analyses between the marker variable and all latent variables showed that correlation coefficients were nonsignificant, which suggested that there was not a substantial amount of common method variance in this study [66]. However, the results of these analyses do not preclude common method bias.

### Data analysis

Relevant statistical packages were used to conduct the analysis, namely, SPSS 24 for descriptive analyses and correlation analyses, and Amos 24 for the path analysis [67]. A *path model* is a diagram relating independent, intermediary, and dependent variables, which may show the mechanism of the relationship between variants. Single arrows indicate causation between extrinsic or intermediary variables and dependent variables, whereas double arrows indicate correlation between pairs of extrinsic variables [68]. Path modelling is an extension of regression modelling and is a kind of structural equation modelling. Path modelling is a much more appropriate approach than typical multiple regressions to illustrate the relationships in complex social sciences contexts because it involves explicit regression, which allows for measurement errors and can estimate the overall goodness of fit of the hypothesized model [69]. Therefore, we constructed a path model based on maximum likelihood estimation to analyse goodness of fit, path coefficients, and coefficients of determination (*R*^2^). Then, the moderating effect of marital status and motherhood were further tested using multiple-group analyses. The statistical values that indicate a good fit are .08 or less for RMSEA, .90 or more for GFI, and .95 or more for CFI and TLI [70, 71].

## Results

### Descriptive statistics

Descriptive statistics and correlations are shown in Table 1 and Table 2, respectively. Among the three types of conflicts, female social workers’ ORC (M = 2.71) was at the highest level, and FWC (M = 1.33) was at the lowest level; compared with their cynicism, female social workers’ exhaustion (M = 1.42) was higher.

**Table 1:** Descriptive statistics of the key study variables

	Mean/No. (Percentage)	SD	Kurtosis	Skewness
Depression	37.96	11.95	3.64	1.71
Family-to-work conflict (FWC)	1.33	0.73	0.72	3.84
Work-to-family conflict (WFC)	1.78	0.84	-0.48	1.00
Organizational role conflict (ORC)	2.71	0.59	1.85	-2.43
Exhaustion (EX)	1.42	0.94	1.04	1.05
Cynicism (CY)	0.31	0.50	3.59	3.12
Age	32.52	8.70	/	
Married	573 (47%)	0.50	/	
No Child	759 (62.9%)	0.64	/	

Note: Age was measured as a continuous variable; marital status was measured as 1= married and 0 = others

No. of child/ren was measured as 0 = no child and 1 = having any number of children

**Table 2:** Correlations

	Depression	FWC	WFC	ORC	EX	CY	Age	Marriage
FWC	.331^**^							
WFC	.368^**^	.560^**^						
ORC	.269^**^	.295^**^	.363^**^					
EX	.535^**^	.326^**^	.483^**^	.369^**^				
CY	.379^**^	.207^**^	.261^**^	.275^**^	.501^**^			
Age	0.019	.060^*^	0.040	0.027	0.050	0.029		
Married	-.162^**^	-.061^*^	-0.053	-.117^**^	-.140^**^	-.104^**^	-.088^**^	
No Child	-.133^**^	-0.017	-0.024	-.092^**^	-.108^**^	-.103^**^	-.085^**^	.694^**^

**. Correlation is significant at the 0.01 level (2-tailed).*. Correlation is significant at the 0.05 level (2-tailed)

In addition, we found that female social workers’ depression positively correlated with FWC (*r* = 0.331, *p* < 0.01), WFC (*r* = 0.368, *p* < 0.01), and ORC (*r* = 0.269, *p* < 0.01). Consistent with previous studies (e.g., Maslach and Leiter [12]), it was also positively correlated with exhaustion (*r* = 0.535, *p* < 0.01) and cynicism (*r* = 0.379, *p* < 0.01) in the workplace. Furthermore, in our studied sample, we found that female social workers’ age did not significantly correlate with their depression and that being married (*r* = -0.162, *p* < 0.01) and having children (*r* = -0.133, *p* < 0.01) were negatively correlated with depression. Specifically, married female social workers who have children reported lower levels of depression.

### Path model

We then tested an initial path model to explore the effects of female social workers’ conflicts on their depression when the mediators of exhaustion and cynicism were excluded (Model 1). The results show that FWC, WFC, and ORC were significantly and positively associated with female social workers’ depression (*β =* 0.16, *p* <.001, *β =* 0.23, *p* <.001 and *β =* 0.14, *p* <.001, respectively). The three kinds of conflicts explained 17% of the variance in depression in total. Thus, Hypothesis 1 were supported.

**Fig. 1:**
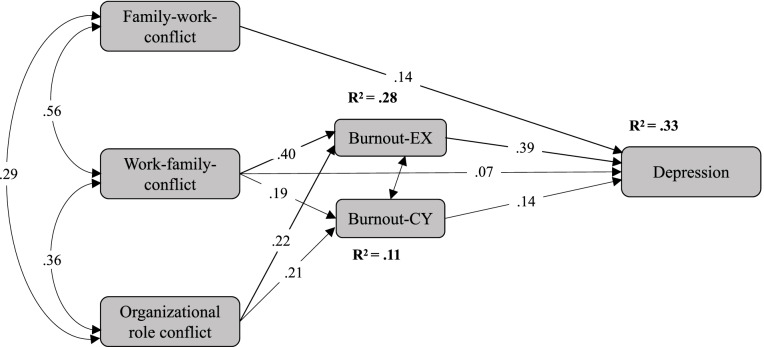
The model of female health care and social service providers' depression from a role conflict perspective

Second, a theory-based structural model was constructed to investigate the mechanism by which three kinds of conflicts influenced female social workers’ depression via exhaustion and cynicism (Model 2). The model fit was good (chi-square = 5.778; degrees of freedom = 3; probability level = 0.123; CFI = 0.999; RFI rho 1 = 0.979; TLI rho 2 = 0.990; RMSEA = 0.028). As a theoretically integrated model, both FWC and WFC were significantly and positively associated with depression (*β =* 0.14, *p* <.001 and *β =* 0.07, *p* <.05, respectively), and ORC was not significantly related to depression. In addition, WFC and ORC were both significantly and positively associated with exhaustion (*β =* 0.40, *p* <.001 and *β =* 0.22, *p* <.001, respectively), while WFC and ORC were both significantly and positively associated with cynicism (*β =* 0.19, *p* <.001 and *β =* 0.21, *p* <.001, respectively), as shown in Fig. 1. Female social workers’ role conflicts, exhaustion and cynicism explained a total of 33% of the variance in depression. Thus, Hypothesis 2 were supported.

However, the significant relationship between ORC and depression became nonsignificant (dropping from *β =* 0.14, *p* < .001 in Model 1 to *β =* 0.00*, p* = .274 in the integrated Model 2), thus indicating the possible presence of a full mediating effect. In addition, the significant relationship between WFC and depression became less significant (dropping from *β =* 0.23, *p* < .001 in Model 1 to *β =* 0.07, *p* < .05 in the integrated Model 2). Because the direct effect of WFC and depression and the effect of ORC and depression become less significant and nonsignificant, respectively, we used 2,000 bootstrapping samples to test for potential mediating effects (95% CI bias-corrected percentile method) [72]. We found that exhaustion and cynicism fully mediated the effects of ORC on depression. The indirect effect (0.116, p < .01) was statistically significant, whereas the direct effect was zero. However, exhaustion and cynicism partially mediated the relationship between WFC and depression. The indirect effect (0.182, p < .01) was statistically significant, whereas the direct effect was not (0.068, *p* < .05). Thus, Hypotheses 3, 4 and 5 were partially supported. Specifically, FWC only had a direct effect on depression, ORC had only an indirect effect on depression, and WFC had both direct and indirect effects on depression.

### Moderation effect of marriage and having child/s

**Fig. 2:**
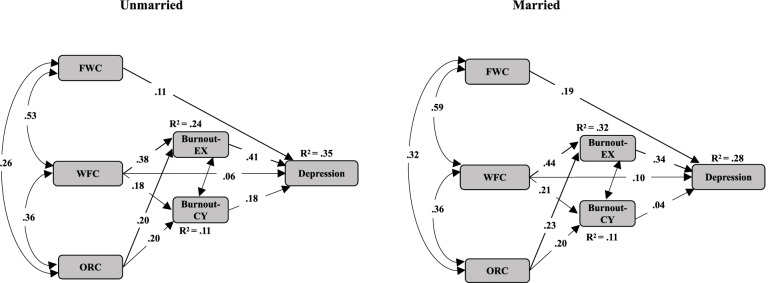
The moderating effect of marriage on female service providers' depression

A multiple-group analysis was conducted to examine the potential moderating effect by adding the moderator of female social workers’ marital status (Model 3; Married, n = 573 vs. unmarried, n = 646). The model fit remained good (X^2^*/df* = 2.528; CFI = 0.996; RFI rho 1 = 0.958; RMSEA = 0.025), indicating that marital status may have played a moderating role in the relationships between cynicism and depression, as indicated by the z-scores (2.17, *p* < .01). Specifically, the positive effect of the relationship between female social workers’ cynicism and depression was more than three times stronger in the unmarried female social worker group (β = 0.18, *p* < .001) than in the married group (β = 0.04, p = .310), as shown in Fig. 2. Thus, Hypothesis 6 was supported.

**Fig. 3:**
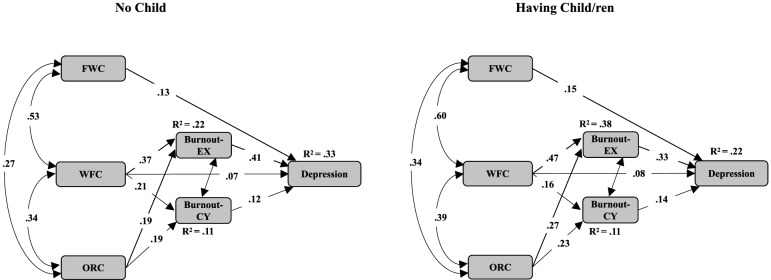
The moderating effect of motherhood on female service providers' depression

A multiple-group analysis was conducted again to examine the potential moderating effect by adding the moderator female social workers’ motherhood status (Model 4; with child/ren, n = 460 vs. without child, n = 759). The model fit remained good (X^2^*/df* = 3.667; CFI = 0.994; RFI rho 1 = 0.939; RMSEA = 0.033), indicating that motherhood status may have played a moderating role in the relationships between WFC and cynicism, as indicated by the *z*-scores (2.277, *p* < .01). Specifically, the positive effect of the relationship between female social workers’ WFC and cynicism was more than twice as strong in the female social worker group with no child (*β =* 0.145, *p* < .001) than in the group with child/ren (*β =* 0.07, *p* < .001), as shown in Fig. 3. Thus, Hypothesis 7 was not supported.

## Discussion and implications

Drawing on conservation of resources theory, this study examined the relationship between different types of role conflicts and depression faced by female social workers, and found the mediating role of burnout and the moderating role of marriage and motherhood. First, the results support the hypothesis that FWC and WFC are directly and positively associated with the depression of female health care and social service providers. It has been previously observed that in addition to workload, employed women have to address family needs (e.g., children care, housework), resulting in their genuinely suffering more distress than men [11]. Since the traditional gender division of labor assigns more housework to women, female health care and social service providers face a stronger work-family conflict. In addition, family needs directly lead to female health care and social service providers’ depressive symptoms.

Second, we found that burnout (exhaustion and cynicism) fully mediated the effects of ORC on depression and partially mediated the effects of WFC on depression. The present work thus echoes previous findings that burnout was a full mediation of the relationships between work-related pressure and depression symptoms among consultants [41] and teachers [15]. Specifically, FWC had only a direct effect on depression, ORC had only an indirect effect on depression, and WFC had both direct and indirect effects on depression. This appears to align with the work outlined by Amstad et al. [45], arguing that work interferences with family were more strongly associated with work-related than family-related outcomes, and family interferences with work were more strongly associated with family-related than work-related outcomes. Burnout is an important factor associated with work-related role conflict and depression among health care and social service providers.

Third, as theoretically tested, FWC was directly associated with the depression of female health care and social service providers, whereas WFC largely had an indirect but significant effect on their depression. A possible explanation could be that family stress affects depression in female health care and social service providers more directly and profoundly. However, descriptive statistics showed that married female health care and social service providers who have children reported lower levels of depression. A multiple-group analysis further indicated that family resources may play a moderating role in the conflict-burnout-depression link and that being unmarried and having no child were risk factors for depression in female health care and social service providers. This reflects the paradoxical principle of conservation of resources theory that resource loss and resource gain occur simultaneously for people facing stressful events and daily stressful situations [22]. In conditions of high losses, efforts that result in small gains may elicit positive expectancy and hope, and reinforce further goal-directed efforts [73]. Therefore, marriage and motherhood may act as “family clientelism” for female health care and social service providers who are married and have children and that “family’s resources or capabilities allow it to thrive in the face of significant risk” [74]. On the one hand, family clientelism has negative consequences on married and childbearing female health care and social service providers, as FWC is directly associated with their depression. On the other hand, through women’s insertion within the institution of marriage and motherhood, the reciprocal exchange relationship established that the psychological wellbeing of these women increased, with less cynicism and less depression.

Based on COR theory, stress occurs when people fail to gain central or key resources (e.g., marriage, child) after significant effort, which can trigger burnout and depression [21, 22]. Previous studies indicate that marriage acts as a protective factor in mitigating psychological distress [19], and in Chinese society, family recognition and support for one’s career are highly valued [75]. For unmarried and infertile female health care and social service providers, they have not yet entered marriage and motherhood as expected by social norms, this “step out of line” means they do not enter the asymmetric relationships of family clientelism. Consequently, unmarried and infertile female health care and social service providers have fewer family resources than married women with children when they face significant work-related risks (role conflicts and burnout) and are more vulnerable to depression symptoms.

Thus, a further explanation for the higher level of depression among female health care and social service providers who were unmarried and had no children is that not only do they have difficulty accessing resources of marriage and family compared to married female workers with children, but their unmarried and childless status may even exacerbate their loss of resources in a male-dominated culture that emphasizes women’s role as wives and mothers. Based on COR theory, a possible explanation for the higher depression level of female health care and social service providers who were unmarried and had no children might be that they were faced with a more profound threat and greater depletion of valued resources than those who were married and had a child. Through an analysis of the causes of depression among female health care and social service providers, we argue that the patriarchal family structure reproduced itself as an embodied, internalized mechanism through the depression of female health care and social service providers, perpetuating sex-role stereotypes in which the family role is primary for women and work roles are secondary [19, 56]. The present work is consistent with the feminist view of depression, which has been quite successful in its accounts of how societal factors build women’s depression [8, 9].

### Implications

The findings of the study highlights the importance of having organizational policies that reduce the work-family conflict and ORC experienced by female health care and social service providers to increase their mental wellbeing. This study supports the existence of two key avenues for reducing depression: burnout and family. This provides managers with evidence to foster an organizational culture conducive to preventing burnout and depression among female health care and social service providers. The finding that burnout is a significant factor associated with work-related role conflicts and depression among female health care and social service providers suggests that managers can increase awareness of burnout issues, and design more effective strategies to lessen and prevent work-related stress.

It is critical for managers and other organizational stakeholders to know how to foster supportive policies between work and family. Such labor policies should also be placed in the context of social work, particularly in a context where the workforce is feminized and where conflicts may arise within and between disciplines. Work-family-friendly work policies have inspired a very important and rapidly growing line of research [18, 76], policymakers and managers can help social workers develop effective strategies for coping with the demands emanating from their work and family domains, such as fostering supportive relationships at work and home, facilitating effective and timely communication in a trusting environment with family members, and setting clear expectations and setting aside ‘me’ time [18]. Organizational policies, including day-care centres for children, homecare for sick and elderly relatives, and flexible work time for workers, would be helpful.

We suggest that a supportive work environment that values gender equality may help female health care and social service providers manage role conflicts better and promote their mental health. On the one hand, female health care and social service providers should be encouraged to enhance bonds with their own families, develop family resilience and seek family resources. On the other hand, we emphasize that by increasing various aspects of organizational support, female health care and social service providers could be more liberated to choose work life as they like rather than relying on marriage and motherhood to receive non-work resources. Drawing on our findings, our implications for female health care and social service providers who experienced depression and sought feminist therapy [77] emphasize an exploration of their inner resources and capacity for self-care, self-healing and transcending sex-role stereotyping. These policies would make a real difference to reduce burnout and depression of female health care and social service providers and may increase the service quality they provide.

## Limitations

There are limitations to our study. First, the study was limited by the method of collecting cross-sectional data, which made it difficult to distinguish the causal mechanisms between role conflicts and depression as well as the specific mechanisms by which burnout plays a mediating role in the role conflict-depression relationship. Second, the present study was exclusively based on a self-report inventory rather than a diagnostic interview and was thus not immune to common method bias. We chose this method based on previous data demonstrating that anonymity is necessary to accurately ascertain depressive symptoms among medical professionals. Nonetheless, it would be important to validate these findings using in-person diagnostic interviews. Third, as previous studies have indicated that the personal vulnerability characteristics of people who enter the profession may be associated with a high level of burnout and depression, this study did not consider personality factors [13, 78]. Finally, previous studies have shown that other health care and social service providers who are also heavily feminized (e.g., occupational therapy, physical therapy, pharmacy, nursing) have a high rate of depression [2]. However, due to our data limitations, they were not included in this research. In future studies, a larger and more representative sample should be used.

## Conclusion

This study identified the relationships between role conflicts, burnout and depression among Chinese female health care and social service providers and provided a better understanding of the factors that may prevent depression. These findings highlight the importance of family resources and work-family-friendly policies to help female health care and social service providers experiencing role conflicts and burnout avoid suffering depression symptoms.

## Data Availability

The data that support the findings of this study are available from East China University of Science and Technology (ECUST) but restrictions apply to the availability of these data, which were used under license for the current study, and so are not publicly available. Data are however available from the authors upon reasonable request and with permission of East China University of Science and Technology (ECUST).

## Abbreviations

FWC: Family-to-Work Conflict
WFC: Work-to-Family Conflict
ORC: Organizational Role Conflict
EX: Exhaustion
CY: Cynicism
